# Editorial: Women in Avian Physiology: 2022

**DOI:** 10.3389/fphys.2022.1086815

**Published:** 2022-11-21

**Authors:** Sandra G. Velleman, Francesca Soglia

**Affiliations:** ^1^ Department of Animal Sciences, The Ohio State University, Columbus, OH, United States; ^2^ Department of Agricultural and Food Sciences, University of Bologna, Bologna, Italy

**Keywords:** avian, genetic selection, muscle physiology, neurobiology, pathology, reproduction

The Women in Avian Physiology Research Topic is part of an inclusive Frontiers series across all sections focused on Women in Physiology. The purpose of the series is to showcase physiological research of women and to highlight their achievements. Submissions were welcomed covering all areas of Avian Physiology. Submissions were encouraged from early career researchers and/or where the lead/last author were females. A total of 12 submissions were accepted for publication in this Research Topic covering the areas of: 1. Skeletal Muscle Physiology and Meat Quality; 2. Female Reproduction; 3. Pathobiology; 4. Genetic Selection; and 5. Neurobiology.

## Skeletal muscle physiology and meat quality

Three contributions addressed skeletal muscle physiology and/or meat quality by Swanson et al., Bordini et al., and Xu et al. Swanson et al. reviewed phenotypic plasticity to environmental variation focusing on metabolic rates and skeletal muscle physiology in wild birds. The ultrastructural plasticity of skeletal muscle with regard to thermal variation and increased workload was reviewed and correlated with myostatin, Insulin-like growth factor-1, and satellite cell proliferation. Xu et al. examined the effects of temperature and selection for growth on the proliferation, differentiation, adipogenic potential of turkey myogenic satellite cells through frizzled-7-mediated Wnt planar cell polarity (Wnt/PCP) pathway. It was found that thermal stress altered frizzled-7 regulation of the Wnt/PCP pathway in a growth-dependent manner affecting the growth potential of the breast muscle and protein to fat ratio. Bordini et al. contributed an original Research Topic studying molecular pathways and key genes associated with White Striping and Wooden Breast in chickens. Using Weighted Gene Co-expression Network Analysis to identify clusters of co-expressed genes associated with White Striping and Wooden Breast, they found that endoplasmic reticulum stress may underly the inflammatory condition in affected breast muscles and Collagen type IV may have significant role in the events leading to White Striping and Wooden Breast.

## Female reproduction

Hanlon et al. provided a comprehensive review on the roles of 17β-estradiol in non-gonadal tissues and its impact on reproduction in both laying and broiler breeder hens. Estradiol-17β are involved in reproduction, liver metabolism, and medullary bone formation. Thus, this hormone may regulate all aspects of egg formation and hence the timing of estradiol-17β is critical to reproduction which has been altered by genetic selection for intense growth. Mehlhorn et al. investigated how hen line, age and housing affect estradiol-17β on egg laying performance. High performance hen lines had higher estradiol-17ß concentrations compared to low performing hens. Regardless of line, maximal estradiol-17ß concentration was measured at their 49th to their 51st week of age. Furthermore, cages hens had highest estradiol-17β levels compared to floor housed hens. We could show that laying performance is strongly linked with estradiol -17ß concentration. This concentration changes during laying period and is also influenced by the housing system.

## Pathobiology

Use of an oral-killed chitosan nanoparticle *Salmonella* vaccine (as an alternative to conventional *Salmonella* poultry vaccines) was shown to decrease *Salmonella* enterica serovar enteritidis load in immunized broiler chickens by Acevedo-Villanueva et al. Shanmugasundaram et al. contributed an original Research Topic demonstrating that subclinical doses of combined fumonisins and deoxynivalenol (mycotoxins contaminating poultry diets) predispose *Clostridum perfringens* inoculated broilers to necrotic enteritis, an economically important disease negatively impacting digestion and absorption of nutrients in broilers.

## Genetic selection

Two contributions addressed the effects of genetic selection. Bernardi et al. reported on chemerin as a possible genetic selection tool for embryo survivability pending larger scale testing. Research on how the somatotrophic axis has changed with commercial growth selection and its relationship to increased growth was reported by Vaccaro et al. They found the expression of insulin-like growth factors 1 and 2 was greater in the modern commercial chicken and maybe linked with increased breast muscle growth and overall muscle accretion. In broilers divergently selected for ultimate pH, it was found by Beauclercq et al. that there is an association between serum lipid profile, ultimate pH and meat quality. Furthermore, since ultimate pH can be obtained on live birds, it may be a useful marker in genetic selection.

## Neurobiology

Loveland et al. contributed a perspective paper on how inversion variants can affect neural circuitry. This review covered how behavior polymorphisms can evolve from genetic inversions, especially when inversions are associated with sets of genes involved with hormonal regulation. Primary focus was on the three-morph system of the ruff (*Calidris pugnax*), two alternative morphs (Satellites and Faeders) each with distinct behaviors and low circulating testosterone that is genetically determined by an inverted region on an autosomal chromosome. Franco et al. examined the bird sense of sight and found that broilers raised under blue light were more hyperopic than those raised with white light. The blue light reared broilers had better spatial vision and higher success in selecting the right feeder.

